# The evaluation and planning method of Spanish sport and physical activity instructors: A comparative study across gender, age, level of studies and work experience

**DOI:** 10.1371/journal.pone.0180228

**Published:** 2017-07-06

**Authors:** Beatriz Bernabé, María Dolores González-Rivera, Antonio Campos-Izquierdo

**Affiliations:** 1 Faculty of Sport and Physical Activity Sciences–INEF, Technical University of Madrid, Madrid, Spain; 2 Faculty of Medicine and Health Science, University of Alcalá, Madrid, Spain; University of Westminster, UNITED KINGDOM

## Abstract

The purpose of this study is to investigate the planning and the evaluation of Spanish sport and physical activity instructors as well as to analyze and compare the two variables in terms of their gender, age, level of studies and work experience. This research falls inside the quantitative type methodology of descriptive cut through standardized interview using the standardized questionnaire: “Human resources of sport and physical activity”. It analyses the situation and performance of people working in functions of sport and physical activity. The questionnaire was completed by 600 sport and physical activity instructors from Spain. Key results revealed that 48.0% of them plan their classes and 58.17% assess. The study also found male university graduates between the ages of 60 and 70, with 10 years of experience or more spend the most time on planning and assessment. Daily classroom observation was the tool which physical activity and sport instructors used the most, followed by execution tests. The lesser used tools were theoretical knowledge exams, diaries and the personally created tests, across all of the variables.

## Introduction

The planning and evaluation that a sport professional performs are essential to promote the success of their jobs [1]. That is why many authors [2–5] have seen the teaching intervention field of human resources of physical activity and sport as an important source of study. The majority of these studies have been conducted in English-speaking countries. Consequently, there is a need to examine this topic outside these countries to provide a more global understanding [2–5]. Beginning with this context, this research is focused on studying the professional and teaching intervention (planning and evaluation) of sport and physical activity instructors in Spain and it is also focused on comparing it in terms of their gender, age, level of studies and work experience.

The recognized profession of sport and physical activity instructor [6], [7] is a resource for the general public. Instructors are specialized in instructing sport and physical activity outside of the educational system [8]. The sample of this research focuses on the sports instructor specialist in sport and physical activity formation. This type of instructor performs the functions of instruction, learning and sports initiation not focused on competition, except for competitions within the program of sport in school events or competitions of recreational character [6], [7]. In the research of Bernabé, Campos-Izquierdo, and González [9] it is stated that in Spain, instructing sports and physical activities is considered a working profession that differs from those functions of a coach and a physical education teacher. A sport and physical activity instructor is defined as someone who can teach a variety of sports or physical activities in an academic setting performed by all types of people and in any facility. It differs from teaching physical education because it is done outside the formal education system. It also differs from coaching because the athletes do not compete. In Martínez´s [10] study, 33.7% of sport workers interviewed worked as sport and physical activity instructors. In Campos-Izquierdo´s [11] study the results were similar with 20% of the workers related to the field of sport working as sport and physical activity instructors. Such high percentages show that in Spain, sport and physical activity instruction is one of the occupations in which most sport professionals work in. To know the real situation of this profession and achieve better results, it is very important to carry out research of this type.

Planning is a reflective function of the sport and physical activity instructor. It consists of organizing the contents of classes and teaching interventions in a flexible and systematic way, fulfilling the objectives, in order to anticipate a future plan of effective action [12]. On the other hand, evaluation is characterized by the need to collect information on performance at different times. Evaluation can be carried out with varied instruments in order to assess the observations according to established criteria and to reach decisions on the fundamental issues of the teaching and learning processes [13].

Coaches´ planning and training assessment have been thoroughly studied [14], [15]. Physical education teachers have also been studied on their planning and training assessment [16], [17]. Meanwhile, other authors like Kravitz [18] investigated the professional and teaching intervention of personal trainers and fitness trainers. But there has been minimal research focusing on sport and physical activity instructors and the research that has been conducted has been superficial and based on methodology [19]. In regards to assessment, in most cases the investigations are exclusively about the evaluations carried out by the coaches exclusively. This is most commonly examined at the university level in specific sports, such as basketball or football. [20]. Despite this tendency, there is research on the evaluation of physical education teachers as well [16], [21]. However, the literature pertaining to the evaluation carry out by sport and physical activity instructors is almost nonexistent. In addition, there are no studies in which the professional and teaching intervention is related with the emerging variables of gender, age, level of studies or work experience. Therefore, it is highly relevant to analyze sport and physical activity instructors by taking into account these variables, while making sure that all parts of the country are well represented.

Consequently, this article covers a wide area regarding the professional and teaching intervention of the sport and physical activity instructors, taking into account the previously mentioned variables, and it is based on and compared with the existing literature on the other sports professions.

Planning is one of the most important functions to properly develop a sport class [22]. All the coaches in Pérez [15] investigation planned the whole season taking into account different factors like results, athletes and sessions. Planning needs to be an ongoing process. Coaches frequently revised and adjusted the originally created program to the emerging conditions [14]. According to our colleagues [4], it is necessary to embrace long-term planning in all aspects that can influence the training of athletes. Moreover, structuring and planning have been reported as coping strategies [15]. Finally, in the teaching process evaluation has always remained an essential step. It is necessary to allow procedures and outcomes to be constantly revised [3]. But the procedures or outcomes are not the only ones to be assessed. The athletes require a lot of feedback [2], [15]. They need to be corrected and stimulated regularly to improve performance and feel comfortable practicing the sport [23]. Sport workers also need to evaluate themselves [20].

Therefore, having seen the need for studies on the professional and teaching intervention of sport and physical activity instructors, the specific objectives of this study are to:

1. Meet and study the planning, evaluation and assessment tools carried out by sport and physical activity instructors.
2. Analyze and compare the planning, evaluation and assessment tools of sport and physical activity instructors in terms of their gender, age, level of studies and work experience.

This will not only add to the depth of existing literature in sport professions but it will also contribute to greater understanding and knowledge of the teaching and professional intervention of Spanish sport and physical activity instructors.

## Methodology

The methodology followed in this investigation was descriptive [24] and the procedures followed were the ones in a sectional survey [25].

### Respondents

Respondents were 600 current sport and physical activity instructors of Spain [8].

Out of the 600 respondents 66.83% were male and 33.17% were female (Table 1) [8]. The participants’ ages ranged from 16 to 70 with a mean age of 30.36 (SD = 9.43); 31.34% from 16 to 24, 58.50% from 25 to 44 and 10.16% from 45 to 70 (Table 1) [8]. According to existing research, these percentages are an accurate reflection of the overall gender and age distribution among sport and physical activity workers in Spain and other countries. Previous studies on sport and the labour market have indicated that the sport and physical activity sector is an important source of employment for youth and women [26], [27]. Consequently, in this study there is an elevated number of women and young professionals compared to other sports professions like coach [12], [13]. In Spain the working age begins at age 16 [28]. Regarding the level of studies, the sample was composed of 228 (38.0%) sport and physical activity instructors who had completed a university degree, 145 (24.16%) who had completed vocational programs, 217 (36.17%) studied secondary school and 10 (1.67%) who had studied primary school or less. Concerning work experience, the sample was composed of 406 (67.67%) sport and physical activity instructors who had less than ten years of experience and 194 (32.33%) who had ten years or more of experience.

**Table 1:** Demographics of the Spanish sport and physical activity instructors (N = 600).

	N	%
**Gender**		
Male	401	66.83
Female	199	33.17
**Age**		
16–19	31	5.17
20–24	157	26.17
25–29	157	26.17
30–34	98	16.33
35–39	61	10.17
40–44	35	5.83
45–49	22	3.67
50–54	24	4.00
55–59	10	1.67
60 or more	5	0.82
**Level of Studies**		
University graduate	228	38.00
Vocational program	145	24.16
Secondary school	217	36.17
Primary scool or less	10	1.67
**Work Experience**		
Less than 10 years	406	67.67
10 years or more	194	32.33

In addition, the sample was made up of sport and physical activity instructors representing three different entities: 142 (21.95%) of sport and physical activity instructors working in a public organization, 330 (51%) working in a private company and 175 (27.05%) working in a sports club.

Since it is an infinite or very large population, and the study is working with a confidence interval of 95.5%, and if it is assuming the population variance in the worst case of p = 50%, then q = 50%, the margin of error sampling allowed would be +2% [29].

### Data collection

A standardized interview questionnaire PROAFIDE: Human Resources of physical activity and sport was used. It was validated [25] and it analyzes the situation and performance of people working in functions of physical activity and sport [30]. To ensure content validity, the following measures were taken: expert opinion provided by 16 independent experts, a discussion group composed of national and international experts, as well as a pilot study utilizing the questionnaire conducted in all regions of the Spanish peninsula [30].

The final version of the PROAFIDE questionnaire consisted of 57 closed questions, divided in five sections: socio-demographic characteristics, sport and physical activity functions, professional performance in specific occupations, work characteristics, and training characteristics [30]. From the PROAFIDE, the items related to the objectives of this study were selected. The three items were to determine: (1) if they have a detailed written plan, (2) if they assessed while they were teaching and (3) what tools they used to evaluate. For the purpose of this investigation the interaction of these items was studied in relation to closed questions that collected specific demographic, educational and labor data (i.e. gender, age, work experience and level of studies).

Those sport and physical activity instructors selected were working in functions of sport and physical activity in all provinces and regions of Spain [8]. A probabilistic multi-stage sampling was employed to select the participants [29]. The groups were stratified by: region, province, municipality, sport facility, and subject to be interviewed. Proportional stratification according geographical area allowed interviewing a maximum of two people in each sports facility [8]. When the interviewers visited the facility, they randomly invited a maximum of two sport and physical activity instructors to participate in the study [8]. The surveys were conducted by 19 interviewers who were primarily university graduates with degrees in Science of Physical Activity and Sport. The interviews were conducted face to face with all the sport and physical activity instructors selected, providing the participants an opportunity to ask any questions they may have had [31], [32]. The respondents were informed that all the information would be kept confidential and that they could withdraw from the study at any time. All interviewers completed an interviewer training seminar which was led by the principal researcher at the Polytechnic University of Madrid. In this training seminar, they were provided with detailed explanations of the procedures to be followed for collecting information, strategies for effective interviewing, and given the opportunity to conduct mock interviews. The surveys lasted 15 minutes and took place at the facilities where the participants were working in [30].

### Data analysis

A univariate and bivariate descriptive analysis has been performed as well as an inferential analysis with Crosstabs Commands that included Pearson χ2 value and significance, and the Phi correlation coefficient. Data analysis was performed after the computer data was tabulated and mechanized, using the statistical package SPSS for WINDOWS (19.0 V).

### Ethical clearance

An ethical clearance was obtained from the ethical commission of the Technical University of Madrid. The commission validated the objective of this project and the methodology. The Law for the Protection of Data was satisfied and fulfilled, not only during the planning, but also during the project. The data were analyzed anonymously.

## Results

### Planning

As seen in the planning row in Table 2, less than half of the sport and physical activity instructors planned their sessions ($\bar{x}$ = 48.0%). Male sport instructors planned a little bit more than females ($\bar{x}male$ = 48.4% and $\bar{x}female$ = 47.2%) (x^2^ = 10325; p < 0.01; Ф = 792). If we refer to age, sport and physical activity instructors who planned the most were the ones from 60 to 70 years ($\bar{x}$ = 60%) (x^2^ = 31880; p < 0.01; Ф = 0.220). As the age of sport and physical activity instructors´ increases, they planned more sport instructors got older they planned more. Along the same line, sport and physical activity instrutorswith more experience also planned more ($\bar{x}$ = 50.5%) (x^2^ = 10062; p < 0.01; Ф = 0.420). In terms of level of studies, sport and physical activity instructors who planned the most were the university graduates, followed by those who have studied a vocational program (see Table 3) (x^2^ = 26994; p < 0.01; Ф = 0.205).

**Table 2:** Planning, assessment and assessment tools used by sport and physical activity instructors (N = 600).

		Percentages
**Planning**	No	52.00
	Yes	48.00
**Assessment**	No	41.83
	Yes, but not regularly	24.67
	Yes, regularly	33.50
**Assessment Tools**	Daily classroom observation	49.60
	Standardized test/ battery tests	8.30
	Execution tests	30.21
	Personally created test	5.73
	Diary	5.33
	Theoretical knowledge exam	0.83

**Table 3:** Planning, evaluation and evaluation tools of sport and physical activity instructors with regard to gender, age, level of studies and experience (N = 600).

		Gender		Age				Level of Studies				Experience	
		Male	Female	16–29	30–44	45–59	60–70	University graduate	Vocational program	Secondary school	Primary school and less	< 10 years	≥10 years
Planning*	No	51.6	52.8	58.9	51.8	50.4	40	43.9	51.0	59.8	69.2	53.0	49.5
	Yes	48.4	47.2	41.1	48.2	49.6	60	56.1	49.0	40.2	30.8	47.0	50.5
Assessment*	No	40.4	44.7	55.5	34.5	41.3	20.0	28.9	42.1	56.1	53.8	46.3	34.0
	Yes. but not regularly	25.2	23.6	19.6	23.0	22.1	20	30.7	24.1	17.8	23.1	24.6	23.7
	Yes. regularly	34.4	31.7	24.9	42.6	36.6	60	40.4	33.8	26.2	23.1	29.1	42.3
Assessment Tools**	Daily classroom observation	47.8	53.5	51.8	50.5	37.1	42.9	46.8	52.9	53.0	36.4	51.6	46.3
	Standardized test/ battery tests	8.8	7.0	7.9	8.7	9.7	0	9.4	8.3	6.0	9.1	8.5	7.9
	Execution tests	30.1	30.2	30.7	27.9	33.9	42.9	28.6	28.9	33.6	36.4	29.3	31.5
	Personally created test	6.8	3.5	3.6	6.7	13.0	0	5.4	6.6	5.4	9.1	4.7	7.4
	Diary	5.5	5.2	5.4	5.8	4.8	0	8.3	3.3	2.0	0	5.3	5.6
	Theoretical knowledge exam	1.0	0.6	0.6	0.4	1.5	14.2	1.5	0	0	9.1	0.6	1.3

*Note*. The numbers refer to the mean in percentage (%).

* p < 0,01

**p < 0,05

### Evaluation and assessment tools

More than half of the sport and physical activity instructors assessed their athletes’ performance ($\bar{x}$ = 58.17%). In addition to planning, assessment showed a statistical difference between gender: males assessed performance more than females ($\bar{x}\mathrm{males}$ = 59.6%; $\bar{x}\mathrm{females}$ = 55.3%) (x^2^ = 10029; p < 0.01; Ф = 0.580). In regard to age, sport and physical activity instructors over 60 years assessed performance more often ($\bar{x}$ = 80%) (x^2^ = 33660; p < 0.01; Ф = 0.230) and they preferred to do so regularly. Regarding level of studies, the university graduates performed assessment most frequently ($\bar{x}$ = 71.1%) (x^2^ = 34303; p < 0.01; Ф = 0.303), followed by the sport and physical activity instructors who have completed a vocational program ($\bar{x}$ = 57.9%) (x^2^ = 34303; p < 0.01; Ф = 0.303). Among those who assess, there is a higher percentage of those who do it regularly. As we saw with planning, sport and physical activity instructors assess more as they have more experience ($\bar{x}$ = 66%) (x^2^ = 11591; p < 0.01; Ф = 0.500).

The analysis of the assessment tools in Table 2 and Table 3 indicated that daily classroom observation was the tool which sport and physical activity instructors used the most ($\bar{x}$ = 49.60%), regardless of their gender (x^2^ = 9782; p < 0.05; Ф = 0.353), their age (x^2^ = 13520; p < 0.05; Ф = 0.350), their level of education (x^2^ = 25498; p < 0.05; Ф = 0.408), and their experience (x^2^ = 10021; p < 0.05; Ф = 0.450). The second most commonly used tool was the execution test ($\bar{x}$ = 30.20%). On the opposite end of the spectrum, it is important to note which assessment tools sport and physical activity instructors report using the least. Specifically, the least used tools were the theoretical knowledge exam ($\bar{x}$ = 0.83%), the diary ($\bar{x}$ = 5.33%), the personally created test ($\bar{x}$ = 5.73%) and the standardized test/ battery tests ($\bar{x}$ = 8.30%).

## Discussion

Results of the current study suggested that, on average, only half of the sport and physical activity instructors plan their classes. This is contrary to what has been observed in coaches by several authors [22]. Planning should be considered one of the primary roles of the sport and physical activity instructors [22]. According to Buceta [22], planning is one of the most important functions of the coach. The same results were obtained by Nash, Sproule, and Horton [33] who said that it is vital for coaches to adopt a long-term planning in all aspects of their practice. Along the same line, Vallé and Bloom [20] found that expert coaches drew up a complete plan for the season. Furthermore, planning must be an ongoing process [14]; coaches have to revise the originally created plan and modify it in response to factors related to the game, the athletes, the coach or the context [1], [14]. The fact that detailed structuring and advanced planning are reported as coping strategies also demonstrates the importance of planning. Moreover, planning in advanced is a way of avoiding situations that coaches know are stressful, in addition to enabling them to use time productively [34]. One possible explanation for our results is that the job of sport and physical activity instructor in Spain is undervalued. Since they do not have a high degree they are not able to give an effective class [35] and that makes slightly more than 50% of them think that they do not need to plan their classes. In addition, 37,84% of workers of this investigation are unable to obtain any degree or qualification in sport and physical activity because they have only finished their studies in primary or secondary school, so they do not possess the necessary skills, knowledge and competences required to develop their job properly.

If we analyze the different variables of the planning study, we observe that men and women gave almost the same importance to planning, with males planning slightly more than women. In regard to age, sport and physical activity instructors aged 60 or more were the workers who plan the most, followed by those aged between 45 and 59, 30 and 44, and finally, between 16 and 29. Also, planning increased along with work experience. Based on these results, senior sport and physical activity instructors drew up more complete plans for the season; they need to plan in order to do a better job. That leads us to believe that planning is a very important aspect. The same conclusion was reached by Vallée and Bloom [20]. Sport and physical activity instructors with a university degree are the ones who plan the most. This could be because they have more training and learning experiences. As seen in the results, sport and physical activity instructors in Spain have a very heterogeneous formation. Their training varies from sport and physical activity instructors who are university graduates to people without any university degree.

Assessment is another important step in teaching physical activity and sports. Coaches normally develop coaching strategies through a process of reflection [36]. According to these authors, the reflection process is composed of six components: coaching issues, role frame, issue setting, strategy generation, experimentation and evaluation [36]. Other authors said that coaching should involve five steps: data collection, diagnosis, action planning, implementation of the plan and evaluation [3]. From one model to another, much research has been done and changes have been proposed, but evaluation has always remained an essential step. That is what the Spanish sport and physical activity instructors of this investigation thought because the results showed that more than half of them assess performance. Sport and physical activity instructors understand the need to assess in order to do their job well. Following our colleagues [3], it is necessary to allow procedures and outcomes to be constantly revised and assessed. With respect to gender, male sport and physical activity instructors appear to prioritize evaluation more than female sport and physical activity instructors. In regard to age, sport and physical activity instructors over 60 years old are the ones who evaluated the most followed by those from 30 to 44 years old. This underlines the importance given by senior sport and physical activity instructors to the professional and teaching intervention [20]. Focusing on the level of studies, the sport instructors who assessed more were those with a university degree, perhaps because the degree provides general knowledge of the profession and those sport and physical activity instructors carry out the ethics of the profession. Finally, taking experience into consideration, as in planning, those workers who had more experience assessed more. This result reveals that experienced sport and physical activity instructors develop the strategy of evaluation through a process of reflection and experimentation [36]. Consequently, these results lead us to conclude that evaluation is another key point in teaching a sport. It is important to analyze why Spanish sport and physical activity instructors assess more than plan. Perhaps they believe that planning is not very important and they can prepare activities during the class or perhaps they prefer to assess first and then find out what to do during the class. The problem is that the ongoing process developed by Dorgo [14] is not carried out like that.

Because of the wide range of conduct and behaviors that can be assessed in physical activity and sport, it has been generated a wide range of tools has been generated [21]. In this study, we have seen that sport and physical activity instructors used a variety of tools. As Wright, Atkins, and Jones [5] said, the tools of assessment have to be flexible, with daily classroom observation being the most commonly used tool. Supporting this point, our colleagues [2] said that coaches are like analyzers; they watch for aspects of their athletes and decide to improve their performance. This is also supported by Kidman and Hanrahan [37] who said that the observation should be considered one of the primary roles of the coach. The second most used tool was the execution tests; this tool is highlighted by Díaz [21], as it has been the most used by physical education teachers. Other assessment tools sport and physical activity instructors often use are battery tests or standardized tests. On the other hand, the assessment tools that they used least were the theoretical knowledge exam, the diary and the personally created tests. Possibly, these assessment tools do not accurately measure the skills and qualities of the athletes. Furthermore, sport and physical activity instructors do not ensure improvement capacity by using those tools.

## Conclusion

This article was intended to provide some descriptive insight into the planning and evaluation methods of sport and physical activity instructors in Spain. The purpose of this study was also to analyze and compare the planning and evaluation in terms of the gender, age, level of studies and work experience of sport and physical activity instructors.

The results obtained indicate that little less than half of sport and physical activity instructors plan their classes and more than half of them assess performance and prefer to do so regularly. The results also show that daily classroom observation is the most used tool by sport and physical activity instructors.

There is a need to plan and assess more in order to lead to an effective learning process. The professional and teaching intervention of Spanish sport and physical activity instructors needs to improve in these two aspects. Because the sport and physical activity instructors in this study did not plan and assess enough, it would be good to promote formal education or offer them more opportunities to learn. Also, due to the heterogeneous formation of sport and physical activity instructors in Spain it would seem beneficial if the government drew up a law regulating the professions of sport in Spain.

## Practical application

The professional and teaching intervention is an inherent component of sport and physical activity instructors and needs to be analyzed better. The current study was the first to incorporate the planning and the evaluation with a variety of measures, but future research can follow this path by analyzing why sport and physical activity instructors behave this way. There is a need for continuation of this research in the Spanish context through a qualitative design as a means of addressing some of the limitations noted in the present study.

## Supporting information

S1 Table Demographics of the Spanish sport and physical activity instructors (N = 600). (DOCX)

S2 Table Planning, assessment and assessment tools used by sport and physical activity instructors (N = 600). (DOCX)

S3 Table Planning evaluation and evaluation tools of sport and physical activity instructors with regard to gender and age (N = 600). (DOCX)

S4 Table Planning, evaluation and evaluation tools of sport and physical activity instructors with regard to level of studies and work experience (N = 600). (DOCX)
