# Supplementary material for: The evaluation and planning method of Spanish sport and physical activity instructors: A comparative study across gender, age, level of studies and work experience
Source: PLoS One. 2017 Jul 6;12(7):e0180228. doi: 10.1371/journal.pone.0180228 (PMC5500318; doi:10.1371/journal.pone.0180228)
Supplement: S1 Table — (DOCX) [file pone.0180228.s001.docx]

**S1 Table. Demographics of the Spanish sport and physical activity instructors (N=600)**

|  | **N** | **%** |
| --- | --- | --- |
| **Gender** |  |  |
| Male | 401 | 66.83 |
| Female | 199 | 33.17 |
| **Age** |  |  |
| 16-19 | 31 | 5.17 |
| 20-24 | 157 | 26.17 |
| 25-29 | 157 | 26.17 |
| 30-34 | 98 | 16.33 |
| 35-39 | 61 | 10.17 |
| 40-44 | 35 | 5.83 |
| 45-49 | 22 | 3.67 |
| 50-54 | 24 | 4.00 |
| 55-59 | 10 | 1.67 |
| 60 or more | 5 | 0.82 |
| **Level of Studies** |  |  |
| University graduate | 228 | 38.00 |
| Vocational program | 145 | 24.16 |
| Secondary school | 217 | 36.17 |
| Primary scool or less | 10 | 1.67 |
| **Work Experience** |  |  |
| Less than 10 years | 406 | 67.67 |
| 10 years or more | 194 | 32.33 |
| **Entities they work for** |  |  |
| Public organization | 142 | 21.95 |
| Private company | 330 | 51.00 |
| Sport club | 175 | 27.05 |
| **Nationality** |  |  |
| Spain | 585 | 97.5 |
| Brazil | 1 | 0.2 |
| Argentina | 4 | 0.7 |
| France | 1 | 0.2 |
| Colombia | 1 | 0.2 |
| Portugal | 1 | 0.2 |
| Uruguay | 1 | 0.2 |
| Italy | 1 | 0.2 |
| Dominican Republic | 1 | 0.2 |
| Cuba | 1 | 0.2 |
| Korea | 1 | 0.2 |
| Others | 2 | 0.3 |
