# Supplementary material for: The evaluation and planning method of Spanish sport and physical activity instructors: A comparative study across gender, age, level of studies and work experience
Source: PLoS One. 2017 Jul 6;12(7):e0180228. doi: 10.1371/journal.pone.0180228 (PMC5500318; doi:10.1371/journal.pone.0180228)
Supplement: S2 Table — (DOCX) [file pone.0180228.s002.docx]

**S2 Table. Planning, assessment and assessment tools used by sport and physical activity instructors (N=600)**

|  | | **N** | **Percentages** | |
| --- | --- | --- | --- | --- |
| **Planning** | **No** | 312 | 52.00 |  |
|  | **Yes** | 288 | 48.00 |  |
| **Assessment** | **No** | 251 | 41.83 |  |
|  | **Yes, but not regularly** | 148 | 24.67 |  |
|  | **Yes, regularly** | 201 | 33.50 |  |
| **Assessment Tools** | **Daily classroom observation** | 298 | 49.60 |  |
|  | **Standardized test/ battery tests** | 50 | 8.30 |  |
|  | **Execution tests** | 181 | 30.21 |  |
|  | **Personally created test** | 34 | 5.73 |  |
|  | **Diary** | 32 | 5.33 |  |
|  | **Theoretical knowledge exam** | 5 | 0.83 |  |
