# Supplementary material for: The evaluation and planning method of Spanish sport and physical activity instructors: A comparative study across gender, age, level of studies and work experience
Source: PLoS One. 2017 Jul 6;12(7):e0180228. doi: 10.1371/journal.pone.0180228 (PMC5500318; doi:10.1371/journal.pone.0180228)
Supplement: S3 Table — (DOCX) [file pone.0180228.s003.docx]

**S3 Table. Planning, evaluation and evaluation tools of sport and physical activity instructors with regard to gender and age (N=600)**

|  | | **Gender** | | | | **Age** | | | | | | | |
| --- | --- | --- | --- | --- | --- | --- | --- | --- | --- | --- | --- | --- | --- |
|  |  | **Male** | | **Female** | | **16-29** | | **30-44** | | **45-59** | | **60 -70** | |
|  |  | **N** | **%** | **N** | **%** | **N** | **%** | **N** | **%** | **N** | **%** | **N** | **%** |
| **Planning*** | **No** | 207 | 51.6 | 105 | 52.8 | 203 | 58.9 | 100 | 51.8 | 28 | 50.4 | 2 | 40 |
|  | **Yes** | 194 | 48.4 | 94 | 47.2 | 142 | 41.1 | 94 | 48.2 | 28 | 49.6 | 3 | 60 |
| **Assessment*** | **No** | 162 | 40.4 | 89 | 44.7 | 191 | 55.5 | 67 | 34.5 | 23 | 41.3 | 1 | 20.0 |
|  | **Yes. but not regularly** | 101 | 25.2 | 47 | 23.6 | 68 | 19.6 | 44 | 23.0 | 13 | 22.1 | 1 | 20 |
|  | **Yes. regularly** | 138 | 34.4 | 63 | 31.7 | 86 | 24.9 | 83 | 42.6 | 20 | 36.6 | 3 | 60 |
| **Assessment Tools**** | **Daily classroom observation** | 192 | 47.8 | 107 | 53.5 | 179 | 51.8 | 98 | 50.5 | 21 | 37.1 | 2 | 42.9 |
|  | **Standardized test/ battery tests** | 35 | 8.8 | 14 | 7.0 | 27 | 7.9 | 17 | 8.7 | 5 | 9.7 | 0 | 0 |
|  | **Execution tests** | 121 | 30.1 | 60 | 30.2 | 106 | 30.7 | 54 | 27.9 | 19 | 33.9 | 2 | 42.9 |
|  | **Personally created test** | 27 | 6.8 | 7 | 3.5 | 12 | 3.6 | 13 | 6.7 | 7 | 13.0 | 0 | 0 |
|  | **Diary** | 22 | 5.5 | 10 | 5.2 | 19 | 5.4 | 11 | 5.8 | 3 | 4.8 | 0 | 0 |
|  | **Theoretical knowledge exam** | 4 | 1.0 | 1 | 0.6 | 2 | 0.6 | 1 | 0.4 | 1 | 1.5 | 1 | 14.2 |

*Note.*

* p < 0,01

** p < 0,05
