# Supplementary material for: The evaluation and planning method of Spanish sport and physical activity instructors: A comparative study across gender, age, level of studies and work experience
Source: PLoS One. 2017 Jul 6;12(7):e0180228. doi: 10.1371/journal.pone.0180228 (PMC5500318; doi:10.1371/journal.pone.0180228)
Supplement: S4 Table — (DOCX) [file pone.0180228.s004.docx]

|  | | **Level of Studies** | | | | | | | | **Work Experience** | | | |
| --- | --- | --- | --- | --- | --- | --- | --- | --- | --- | --- | --- | --- | --- |
|  |  | **University graduate** | | **Vocational program** | | **Secondary school** | | **Primary school and less** | | **< 10 years** | | **≥10 years** | |
|  |  | **N** | **%** | **N** | **%** | **N** | **%** | **N** | **%** | **N** | **%** | **N** | **%** |
| **Planning*** | **No** | 100 | 43.9 | 74 | 51.0 | 130 | 59.8 | 7 | 69.2 | 215 | 53.0 | 96 | 49.5 |
|  | **Yes** | 128 | 56.1 | 71 | 49.0 | 87 | 40.2 | 3 | 30.8 | 191 | 47.0 | 98 | 50.5 |
| **Assessment*** | **No** | 66 | 28.9 | 61 | 42.1 | 122 | 56.1 | 6 | 53.8 | 188 | 46.3 | 66 | 34.0 |
|  | **Yes. but not regularly** | 70 | 30.7 | 35 | 24.1 | 38 | 17.8 | 2 | 23.1 | 100 | 24.6 | 46 | 23.7 |
|  | **Yes. regularly** | 92 | 40.4 | 49 | 33.8 | 57 | 26.2 | 2 | 23.1 | 118 | 29.1 | 82 | 42.3 |
| **Assessment Tools**** | **Daily classroom observation** | 107 | 46.8 | 77 | 52.9 | 115 | 53.0 | 4 | 36.4 | 209 | 51.6 | 90 | 46.3 |
|  | **Standardized test/ battery tests** | 22 | 9.4 | 12 | 8.3 | 13 | 6.0 | 1 | 9.1 | 35 | 8.5 | 15 | 7.9 |
|  | **Execution tests** | 65 | 28.6 | 42 | 28.9 | 73 | 33.6 | 4 | 36.4 | 119 | 29.3 | 61 | 31.5 |
|  | **Personally created test** | 12 | 5.4 | 9 | 6.6 | 12 | 5.4 | 1 | 9.1 | 19 | 4.7 | 14 | 7.4 |
|  | **Diary** | 19 | 8.3 | 5 | 3.3 | 4 | 2.0 | 0 | 0 | 22 | 5.3 | 11 | 5.6 |
|  | **Theoretical knowledge exam** | 3 | 1.5 | 0 | 0 | 0 | 0 | 1 | 9.1 | 2 | 0.6 | 3 | 1.3 |

**S4 Table. Planning, evaluation and evaluation tools of sport and physical activity instructors with regard to level of studies and work experience (N=600)**

*Note.*

* p < 0,01

** p < 0,05
